# Combining Gold and Selenium as Emerging Bioactive Compounds: From Synthesis to Therapeutic Potential

**DOI:** 10.1021/acsomega.5c08885

**Published:** 2026-01-23

**Authors:** Katherine Lima Bruno, Patrícia Salvador Tessaro, Mariana F. Soares, Eduardo E. Alberto, Heveline Silva

**Affiliations:** † Laboratory of Bioinorganic Synthesis and Interactions (SIBLab), Department of Chemistry, Institute of Exact Sciences, 28114 Universidade Federal de Minas Gerais, Belo Horizonte, MG 31270-901, Brazil; ‡ Green Chemistry and Organocatalysis Laboratory (GCO Lab), Department of Chemistry, Institute of Exact Sciences, Universidade Federal de Minas Gerais, Belo Horizonte, MG 31270-901, Brazil

## Abstract

This review outlines an overview of selenium-containing gold compounds, framed within the context of gold chemistry. It emphasizes key synthetic strategies and structural features of Au–Se complexes, encompassing both gold­(I) and gold­(III) species. Various classes of selenium ligands are discussed, including selenols, selenoureas, selenones, and selenium-NHCs, among other organoselenium ligands. Despite the relatively limited number of studies, these complexes exhibit diverse biological activities, including anticancer, antimicrobial, and anti-inflammatory effects. Mechanistic evidence suggests that these activities primarily arise from inhibition of thiol- and selenol-containing enzymes (e.g., thioredoxin reductase), disruption of redox homeostasis, or the induction of reactive oxygen species (ROS) formation. The synergistic interplay between gold and selenium centers is crucial for modulating these effects. This review highlights emerging trends in ligand optimization, providing a foundation for the rational design of next-generation selenium-based gold therapeutics.

## Graphical abstract

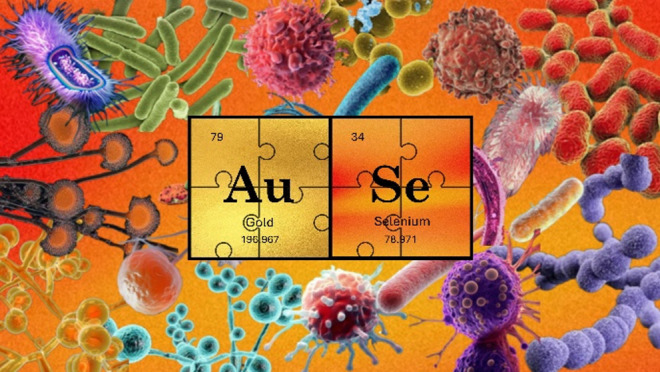

## 1 Introduction

### 1.1 History and Evolution of Gold-Based Compounds

Gold has been used as a metallotherapeutic since ancient times, with its earliest applications reported in China around 2500 B.C.^1^ In modern research, gold­(I) and gold­(III) complexes with organic ligands—whose lipophilicity and functional properties can be tuned—have been reintroduced into clinical medicine to modulate cellular processes involved in infections, rheumatoid arthritis, and cancer.^2^

The first bioactive gold complex was reported in 1890 by the German physician Robert Koch, who discovered that K­[Au­(CN)_2_] was bacteriostatic and was therefore used in the treatment of pulmonary tuberculosis, albeit without substantial therapeutic success.^2,3^ Despite its toxicity, the period from 1925 to 1935 became known as the “gold decade”.^4^ While its efficacy against tuberculosis was limited, the compound showed notable potential in alleviating symptoms of rheumatoid arthritis (RA), effectively reducing joint pain in nontubercular patients receiving gold-based therapy.^1^

This discovery prompted further investigations into gold­(I) complexes, resulting in the development of intramuscular formulations such as Aurothioprol (Allochrysine) 1, Aurothiomalate (Myocrisin) **2**, Aurothiosulfate (Sanochrysin) **3**, and Aurothioglucose (Solganol) **4** for RA treatment. To replace injectable therapies, auranofin (AF) **5**–a triethylphosphine gold­(I) 2,3,4,6-tetra-*O*-acetyl-β-d-glucopyranosyl-1-thiolate complex–was developed in 1970 and approved by the FDA in 1985 for oral administration^5^ (Figure 1).

**Figure 1:**
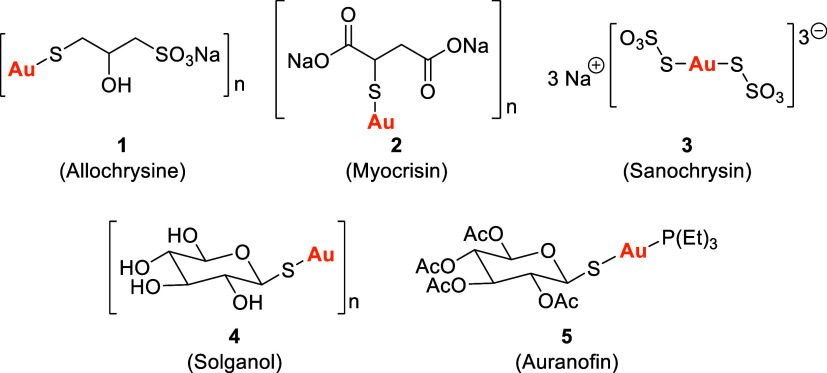
Clinically used antirheumatic gold­(I) compounds. Structures have been drawn according to the PubChem database (https://pubchem.ncbi.nlm.nih.gov/).

In 1979, it was discovered that auranofin **5** inhibited the proliferation of cervical cancer cells (HeLa) in vitro and exhibited antitumor effects in leukemia cells (P388) in vivo. This finding initiated extensive research into structural analogs of auranofin. Since then, linear gold­(I) complexes containing phosphines, sulfur-based ligands (e.g., thiosugars, thionucleobases, dithiocarbamates, and sulfanylpropenoates), *N*-heterocyclic carbenes, and bioactive vitamin K_3_ derivatives (such as azacoumarin and naphthalimide) have demonstrated significant cytotoxic activity, efficiently inhibiting tumor cell growth in vitro.^4^

Although auranofin showed a lower incidence of side effects and was used clinically for many years, it proved less effective in treating RA and went off patent in 1992. Nevertheless, its potential in diverse biological activities has been investigated, including anti-inflammatory, antiviral, antifungal, antiparasitic, antibacterial, antidiabetic, and primarily antitumor effects (Figure 2).^5^

**Figure 2:**
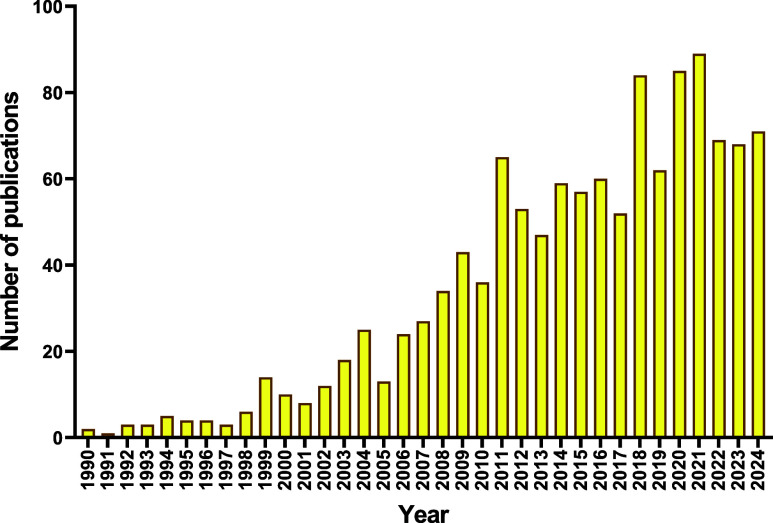
Evolution of Scopus-indexed publications on gold complexes with biological activity (1990–2024).

In this context, gold complexes have gained increasing relevance in therapeutic research, particularly due to their anticancer properties. While anticancer activity remains the most studied aspect of gold medicinal chemistry, anti-infectious effects—targeting parasitic, bacterial, and viral infections—have also received considerable attention. Consequently, gold compounds contribute to understanding mechanisms of action and identifying new therapeutic targets.^6–17^ These advances will guide the development of more effective gold-based drugs for treating cancer, autoimmune disorders, and parasitic infections.

### 1.2 Gold­(I) and Gold­(III)

The cytotoxic effects of auranofin **5** and other gold compounds are mainly due to the Lewis acidity of gold ions, a key factor underlying their reactivity and biological activity. As soft acids, gold­(I) ions (5d^10^) preferentially bind to soft donor ligands. They have a high affinity for sulfur, selenium, phosphorus, and carbene-containing compounds, forming linear dicoordinated complexes such as compounds **1–5** (Figure 1).^18,19^ These ligands are critical for biological activity, enabling gold­(I) complexes to target enzymes and metabolic pathways selectively, trigger oxidative stress, modulate redox signaling, and activate apoptosis in cancer cells and pathogens. This distinctive coordination ability enhances stability and specificity, underscoring their therapeutic potential.^4,6,18^

In contrast, gold­(III) ions, with a d^8^ configuration, favor tetra-coordination in a square planar geometry and tend to bind to harder ligands, such as nitrogen (e.g., complexes **6** and **7**) and, less commonly, oxygen donors, making them isostructural to Pt­(II) compounds like cisplatin (*cis*-diaminedichloroplatinum), **8** (Figure 3).^18–20^ This similarity suggests that gold­(III) complexes may share cytotoxic mechanisms with platinum-based drugs, positioning them as promising candidates for cancer chemotherapy.^21^ However, a significant challenge in the development of gold­(III)-based therapeutics lies in their chemical instability and reproducibility issues under physiological conditions.^22^

**Figure 3:**
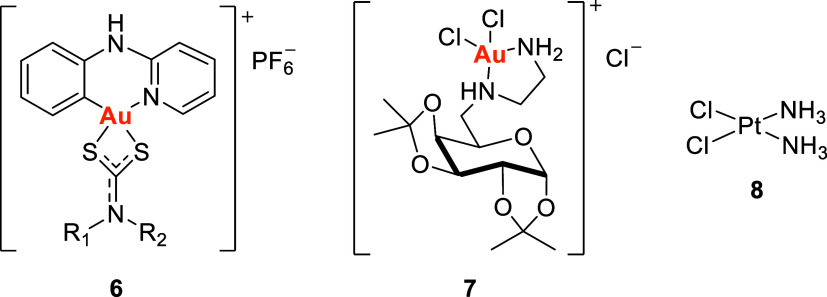
Planar square gold­(III) compounds **6** and **7** and cisplatin **8**. Adapted with permission from ref 23, Copyright 2023 Elsevier, and ref 24, Copyright 2015 Springer Nature.

Gold­(III) compounds are prone to reduction to Au­(I) or Au(0), particularly in the presence of biological reducing agents. This feature compromises stability, reduces cytotoxic activity, and limits bioavailability, while promoting an undesirable metal accumulation in organs. In some cases, Au­(I)/Au­(III) speciation contributes to bioactivity. However, it often leads to rapid ligand exchange and reduction of Au­(III) due to high redox potential. Ligand release or free metal under physiological conditions can cause adverse effects.^22,25^ Despite instability, recent advances in stabilizing Au­(III) oxidation states have renewed interest in organogold­(III) complexes, with ligand exchange and reduction to Au­(I)/Au(0) influencing anticancer efficacy.^25–31^

### 1.3 Gold Targets and the Role of Selenium

The recognition of targets for gold compounds, including selenoproteins, mitochondria, cysteine proteases, and transcription factors, suggests that gold-based drugs could be leveraged for the treatment of various diseases linked to the dysfunction of these targets.^4^ Gold compounds selectively interact with thiol (–SH) and selenol (–SeH) groups in enzymes, targeting parasites, bacteria, and cancer cells. These compounds can also disrupt selenium metabolism, crucial for selenoprotein synthesis.^32–35^ Research has grown substantially on repositioning auranofin and designing new gold compounds for broad therapeutic applications.^36–38^

Selenium is particularly relevant due to its role in antioxidant defense and redox regulation. Selenium-containing compounds, mainly selenomethionine (SeMet) and selenocysteine (SeC), form selenoenzymes such as thioredoxin reductase (TrxR) and glutathione peroxidase (GPx).^39–41^ These enzymes maintain the redox homeostasis and protect biomolecules from oxidative damage.^42–44^ According to Pearson’s HSAB theory, selenium behaves as a soft base, favoring coordination to soft metals like gold.^43,45,46^ Au­(I) has a high affinity for selenium, explaining its reactivity with selenium-based biomolecules. This selectivity translates into inhibition of redox-active selenoenzymes central to cellular homeostasis, such as TrxR and GPx.

The mechanisms behind these activities (Figure 4) involve the TrxR system’s inhibition, composed of the selenoprotein thioredoxin reductase (TrxR) and the thiol protein thioredoxin (Trx), as well as glutathione reductase (GR) and glutathione peroxidase (GPx). These enzymes, located in both the mitochondria and cytosol, play key roles in maintaining cellular redox homeostasis, protecting it against reactive oxygen species (ROS), especially peroxides.^47^

**Figure 4:**
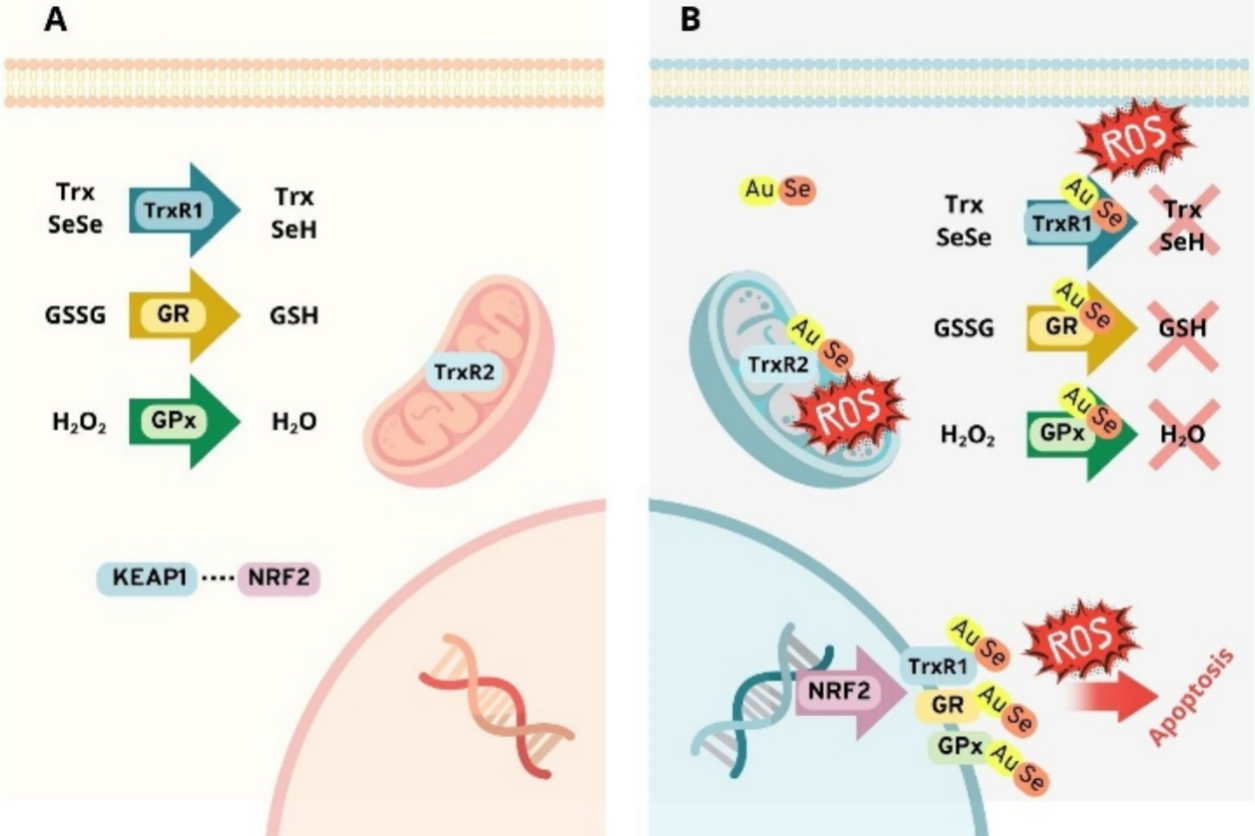
Representation of (A) the redox balance of normal cells and (B) the redox balance with gold–selenium interference driving stressed cells to apoptosis.

The glutathione system operates through GPx, which catalyzes the reduction of peroxides such as H_2_O_2_, using reduced glutathione (GSH) as an electron donor and producing oxidized glutathione (GSSG). The enzyme GR regenerates GSH from GSSG using NADPH, maintaining a functional antioxidant cycle. In parallel, the thioredoxin system is composed of TrxR, which reduces oxidized thioredoxin (Trx-SeSe) to its active form (Trx-(SeH)_2_), also using NADPH as a cofactor.^48^

These two systems operate in a coordinated and partially redundant manner and are widely regulated by NRF2 (Nuclear factor erythroid 2-related factor 2). In situations of oxidative stress, the transcription factor NRF2 is released from its inhibitory protein (KEAP1) and translocates to the nucleus, where it activates antioxidant genes. This compensatory upregulation, reflected in the increased production of TrxR and GPx, underscores the centrality of selenol-containing enzymes as targets for redox modulation, providing a mechanistic basis for their interaction with Au­(I) species.^49^

Due to the strong binding affinity between Au­(I) and selenium–in agreement with Pearson’s principle–Au­(I)–Se complexes interact with selenol-containing enzymes, forming Au-enzyme adducts. This disrupts antioxidant mechanisms, underpinning Au–Se bioactivity.^43,50–52^ Targeted interactions with selenoenzymes produce pronounced cytotoxic effects via GPx and TrxR’s inhibition.

Gold compounds exhibit selective cytotoxicity by targeting selenium residues and thiol groups, thereby impairing cellular peroxide detoxification and promoting intracellular ROS accumulation.^53–56^ This oxidative imbalance induces damage to lipids, proteins, and DNA, ultimately triggering apoptosis or, in some contexts, ferroptosis.^57–59^ The frequent overexpression of TrxR in cancer cells underscores its potential as a therapeutic target.^60^ These vulnerabilities have driven the rational design of gold complexes with enhanced selectivity, notably through the replacement of sulfur with selenium as the coordinating chalcogen. In this way, it is possible to exploit selenium’s unique nucleophilicity, redox properties, and lipophilicity to improve the biological efficacy of gold complexes.

In this context, replacing sulfur with selenium as the coordinating chalcogen in gold complexes has emerged as a promising strategy. Selenium exhibits higher nucleophilicity than sulfur, as expected by its higher acidity (p*K*_a_ RSeH ≈ 5.2 vs p*K*_a_ RSH ≈ 8.3), resulting in more reactive selenolates. Moreover, selenium-containing ligands enhance lipophilicity, which may facilitate interactions with cellular membranes and favor binding within hydrophobic enzyme pockets. These properties collectively expand the potential of Au–Se systems for applications in medicinal bioinorganic chemistry,^43,44^ complementing selenium’s intrinsic biological significance.

Selenium is a trace element of fundamental biological importance, largely due to its incorporation into selenoproteins. In the human body, it occurs in both inorganic forms (such as selenates and selenites) and organic species, primarily selenomethionine (SeMet) and selenocysteine (SeC). Its recognition as an essential micronutrient dates back to the 20th century, when selenium was shown to prevent deficiency diseases such as Keshan disease. Selenium plays a pivotal role in physiological functions, particularly in regulating antioxidant systems, whose homeostasis requires a delicate balance between adequate levels and potential toxicity.^61^

Beyond its essential role in selenoproteins such as thioredoxin reductase (TrxR) and glutathione peroxidase (GPx), selenium is highly relevant in medicinal chemistry due to its superior reactivity compared to sulfur. The selenol group (Se–H) has a substantially lower p*K*_a_ than the analogous thiol, favoring the formation of highly nucleophilic selenolates that coordinate to metal centers such as gold more rapidly and efficiently, providing mechanistic and catalytic advantages. In parallel, organoselenium compounds have been extensively investigated as potential chemopreventive agents, with recent studies highlighting their antioxidant and antitumor activities.^62^

Depending on its chemical form and cellular context, selenium can act as both an antioxidant and a pro-oxidant agent. Inorganic forms tend to exert a pro-oxidant effect on thiol groups, promoting the generation of oxygen-free radicals, while organic forms are more readily metabolized and excreted.^63^ Moreover, excess selenium or reactive selenium species can covalently modify the selenocysteine residue at the active site of selenoenzymes, impairing their catalytic activity, as demonstrated by the Ethaselen-mediated inhibition of thioredoxin reductase 1 (TrxR1).^64^ Conversely, compounds such as sodium selenite have demonstrated relevant chemopreventive properties.^65^

Historically, the development of Au–Se complexes began with simple selenolates, selenoacid derivatives, and other early selenium-based ligands.^42,45,50–52^ More recently, it has progressed to sophisticated ligand frameworks.^46,66–69^ These advances not only expanded the scope of Au-chalcogen chemistry, particularly in the context of Au–Se complexes, but also provided the basis for subsequent investigations into their biological relevance. In particular, anticancer, antimicrobial, and anti-inflammatory applications have emerged as major areas of interest. Nevertheless, while the synthetic strategies leading to these complexes have been thoroughly addressed in previous studies, the present review will specifically focus on their biological activity and therapeutic potential.

Despite this potential, the literature on biologically active gold–selenium (Au–Se) complexes remains limited and relatively recent (Figure 5), especially when compared to the extensive research on gold–sulfur (Au–S) systems. This disparity highlights the urgent need for comprehensive structural, mechanistic, and therapeutic investigations to fully elucidate the Au–Se species behavior.

**Figure 5:**
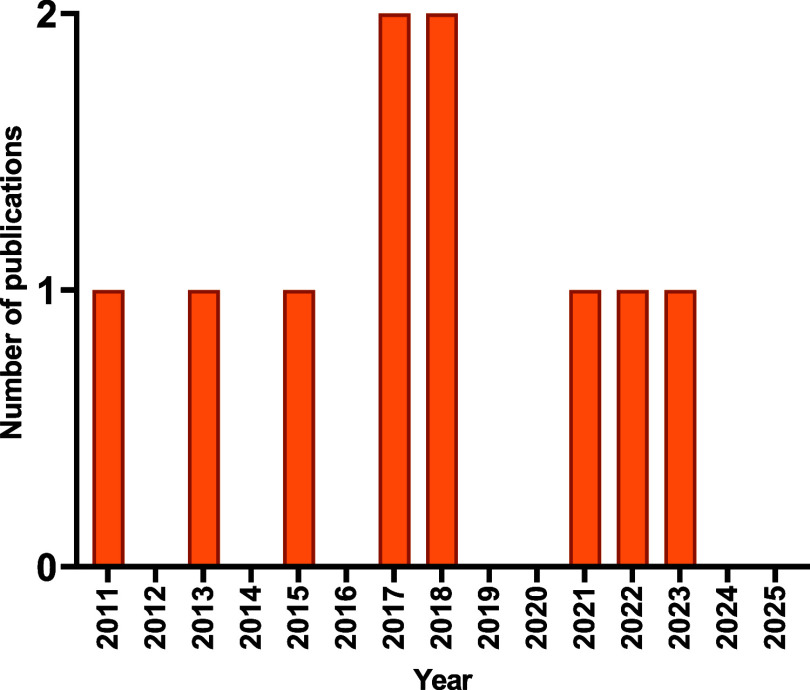
Temporal evolution of Scopus-indexed publications on biologically active gold–selenium complexes (2010–2025).

Notably, the combined use of gold and selenium compounds—such as the coadministration of auranofin (AF) and selenocysteine—has been explored as a strategy to potentiate thioredoxin reductase inhibition, a key therapeutic target in oncological and infectious diseases.^70,71^

### 1.4 Advancement of Gold–Selenium Complexes as Antitumoral and Antimicrobial Agents

Cancer remains one of the most pressing global health challenges due to its biological complexity and the limited efficacy of current therapies. Despite major advances and extensive efforts to develop new treatment strategies, it continues to rank as the second leading cause of death among noncommunicable diseases (NCDs).^72^

Within this scenario, the investigation of gold and selenium compounds as anticancer agents has intensified over the past decade, broadening their therapeutic scope beyond traditional medicinal applications. The anticancer activity of gold complexes is primarily associated with inhibition of thioredoxin reductase (TrxR), resulting in disruption of intracellular redox balance, induction of oxidative stress, and increased production of reactive oxygen species (ROS).^73,74^ In addition to TrxR, other biological targets such as glutathione and mitochondria have been implicated, given their central roles in maintaining redox homeostasis and mitigating oxidative damage.^75–88^ The inhibition of these antioxidant systems leads to elevated ROS levels, triggering apoptosis and ultimately inducing cell death.^89–91^

Building on this redox-based therapeutic concept, selenium compounds are well recognized for their dual redox-modulating behavior and enhanced selectivity toward malignant cells. Their pro-oxidant properties—largely responsible for cytotoxic and anticancer effects—arise from ROS generation, oxidation of protein thiols, and direct or indirect interactions with DNA. Organoselenium compounds induce selective oxidative stress in tumor cells through participation in redox cycles involving glutathione (GSH) and thioredoxin/glutaredoxin (Trx/Grx) systems, thereby amplifying ROS production, disrupting redox homeostasis, and activating apoptotic pathways. Their selectivity stems from the higher uptake of selenium by cancer cells and their intrinsically dysregulated redox environment, enabling targeted cytotoxicity with reduced systemic toxicity.^92^

Beyond their anticancer potential, gold complexes have also attracted increasing attention for their antimicrobial and, to a lesser extent, anti-inflammatory properties. Their distinctive redox reactivity and strong affinity for soft biological nucleophiles allow them to interfere with critical metabolic and enzymatic processes, broadening their therapeutic versatility.^93–96^

Gold–selenium (Au–Se) complexes, in particular, have demonstrated promising antimicrobial activity against a range of pathogens, including *Staphylococcus aureus* (notably vancomycin-intermediate strains) and multidrug-resistant Gram-negative species such as Acinetobacter baumannii, *Pseudomonas aeruginosa*, *Klebsiella pneumoniae*, and *Escherichia coli*. Their mechanisms of action involve irreversible TrxR inhibition, induction of oxidative stress, and impairment of essential cellular functions such as membrane integrity and energy metabolism.^97,98^ In addition, Au–Se complexes have shown activity against the fungus *Candida albicans* and the protozoan parasite *Plasmodium falciparum*.

Although less extensively studied, anti-inflammatory effects have also been reported. Gold complexes have exhibited significant activity in both cellular and animal models, with proposed mechanisms including inhibition of redox enzymes, modulation of antioxidant defenses, and suppression of inflammatory mediators.^99^ To contextualize their therapeutic potential, the following section summarizes recent studies addressing the structural features, biological activities, and mechanisms of action of Au–Se complexes.

## 2 Gold Complexes with Organoselenium Ligands

### 2.1 Gold­(III) and Selenic Acid Derivatives

In the first study reporting the anticancer activity of a gold–selenium complex (2013), Refat and collaborators developed gold­(III) complexes, containing two tails of chelating selenium ions as cofactors for adipic (**9a**) and sebacic (**9b**) acids, that showed cytotoxicity against Ehrlich ascites carcinoma cells (EACC). The incorporation of selenium into the gold–adipic and gold–sebacic complexes (Scheme 1) enhanced cytotoxicity up to 3-fold compared to the parent acids, reducing cell viability from 100% to nearly 0%.^100^

**Scheme 1:**
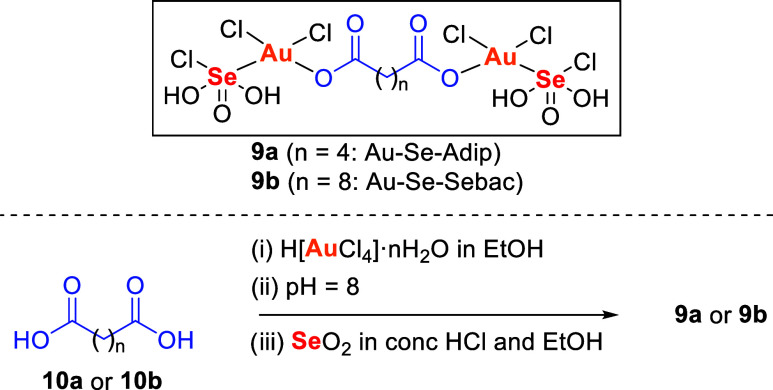
Selenic Acid Derivatives Gold Complexes

These compounds were prepared by the addition of chloroauric acid hydrate (H­[AuCl_4_]·*n*H_2_O), to adipic or sebacic acid dissolved in ethanol. After neutralization of the solution to pH 8 with NH_4_OH, a solution of selenous acid (H_2_SeO_3_), freshly made from selenium dioxide (SeO_2_) in concentrated HCl and ethanol, was added to give **9a** or **9b**.

In the study, biochemical parameters associated with cytotoxic effects, including glutathione-S-transferase (GST) activity, reduced glutathione (GSH) levels, and malondialdehyde (MDA) levels, were evaluated. GST and GSH act as antioxidants in detoxification pathways, protecting cells against oxidative stress. While their upregulation in the presence of free radicals contributes to tumor cell survival, MDA serves as a marker of lipid peroxidation, typically showing higher levels in tumor tissues than in healthy organs, with untreated carcinoma cells or cells treated with reference compounds. The results indicated that compound **9b** enhanced GST activity and increased GSH levels, while also inhibiting MDA formation, compared with untreated carcinoma cells and those treated with other synthesized compounds.

Complexes **9a** and **9b** displayed distinct antibacterial profiles compared with each other and with the reference antibiotic tetracycline. Complex Au-Sebac **9b** demonstrated superior efficacy against Gram-positive strains, notably *S. aureus*, where it achieved an inhibition zone of 22 mm, surpassing tetracycline (20 mm). In contrast, complex Au-SeAdip **9a** exhibited moderate antibacterial activity, with smaller inhibition zones overall, though still comparable to tetracycline for some strains. These findings suggest that the variation in spacing between the carboxylic groups of the dicarboxylic ligands (adipic vs sebacic acid) plays a critical role in the interaction with bacterial targets.

In antifungal assays, both complexes were active against *C. albicans*. Once again, complex **9b** outperformed its analogue, producing an inhibition zone of 19 mm, close to that of amphotericin B (21 mm). Complex **9a**, in contrast, showed lower activity (14 mm). This difference indicates that the sebacic ligand in **9b** may enhance penetration or affinity toward fungal cell wall components. The direct comparison with amphotericin B underscores the therapeutic potential of complex **9b**, whose antifungal activity approaches that of a clinically established drug. Collectively, the results highlight the superior performance of **9b** in both antibacterial and antifungal assays, reinforcing the importance of ligand structure in modulating the antimicrobial activity of Au–Se complexes. Although the precise molecular targets of the antifungal effects were not identified, selenium is likely to contribute through mechanisms involving oxidative stress induction or disruption of membrane integrity.^101,102^

### 2.2 Gold­(I) with Selenium-NHC Derivatives: Selenoureas and Selenones

Gold­(I) complexes bearing selenium-coordinated *N*-heterocyclic carbene (NHC) ligands, commonly referred to as selenones or selenoureas–when a urea backbone is present–represent a significant evolution in Au–Se chemistry.^103^ The first cyclic selenoureas were described by Nolan’s group in 2014, establishing the foundation for subsequent investigations into Au­(I)–Se coordination frameworks.^46^

Building on this, De Franco and colleagues^104^ developed Au­(I) complexes based on NHC-selenoureas (**11a–11h**) to assess their anticancer potential in 2D and 3D cellular models (Figure 6).

**Figure 6:**
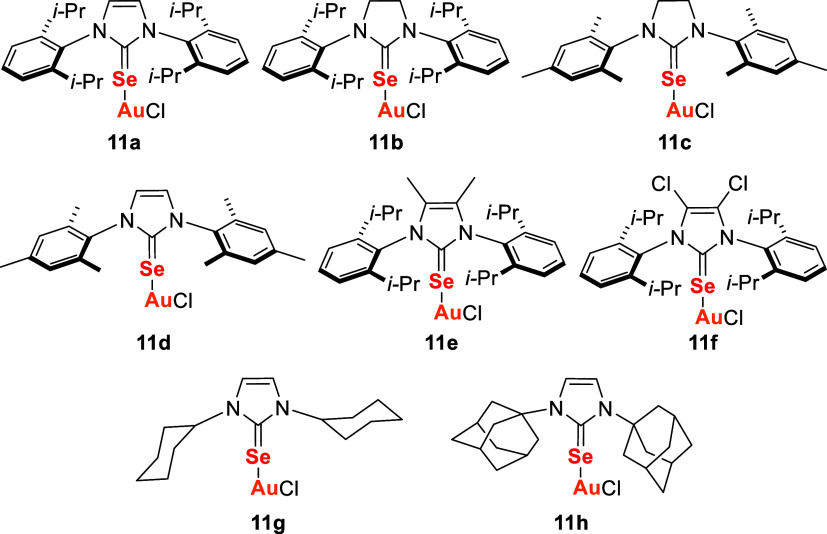
Gold complexes with NHC-based selenourea ligands **11a**–**11h**.

The preparation of the selenourea Au­(I) complexes **11a**–**11h** can be achieved in two steps (Scheme 2). Initially, imidazolium or 4,5-dihydro-imidazolium salts **12a**–**12h** are converted to the corresponding selenourea derivative **13a**–**13h** by treatment with elemental selenium and a weak base (e.g., K_2_CO_3_) in ethanol or acetone. The Au­(I) complexes **11a**–**11h** are then obtained by the reaction between **13a**–**13h** and chloro­(dimethylsulfide)­gold­(I), [AuCl­(SMe_2_)], in acetone.

**Scheme 2:**
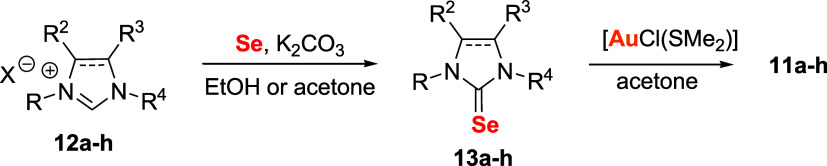
Synthesis of Gold Complexes (**11a**–**11h**) with NHC-Based Selenourea Ligands (**13a**–**13h**)

These complexes showed strong cytotoxicity, comparable to or greater than that of auranofin **5**, and remained active against platinum-resistant and multidrug-resistant cancer cells. They presented IC_50_ values in the low to submicromolar range (8.0–0.3 μM) in several human cancer lines, including LoVo, LoVo-OXP (colon), LoVo MDR, and ovarian adenocarcinoma A2780 and A2780 ADR. In 3D spheroid assays with lung (H157) and ovarian (A2780) cells, their efficacy surpassed that of cisplatin and Ag­(I) derivatives. Mechanistic studies confirmed selective inhibition of thioredoxin reductase (TrxR), with no significant effect on glutathione reductase (GR). In H157 cells, TrxR inhibition disrupted redox balance and mitochondrial activity, leading to apoptosis.

Another study on NHC-based selenoureas was conducted by Seliman et al.,^105^ who evaluated the in vitro cytotoxicity and amino acid interactions of the adduct [Au­(IPr)­(**Se**u)]­PF_6_ **16** (Scheme 3). The complex was synthesized via counterion exchange between **14** and AgPF_6_ in an ethanol/dichloromethane mixture, followed by coordination of selenourea **15**.

**Scheme 3:**
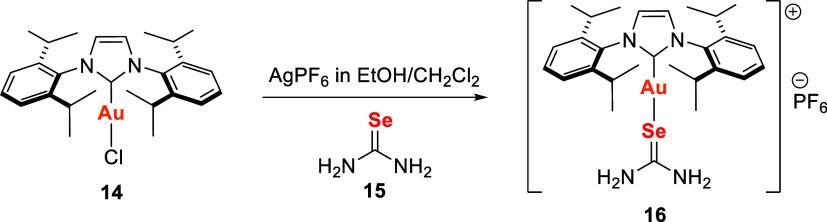
Synthetic Route of Gold­(I) Complex with Selenourea and NHC Ligands, [Au­(IPr)­(Seu)]­PF_6_ **16**

The IC_50_ data indicated that complex **16** was less potent than cisplatin but more active than its precursor [Au­(IPr)­Cl] **14**, suggesting that selenourea coordination enhances the inhibitory efficiency of the gold­(I) center. Electrochemical analysis confirmed interactions between complex **16** and biological thiols, such as glutathione and l-cysteine, evidenced by shifts in redox peaks toward more positive potentials and changes in current intensity.

Subsequent studies by the same group^106,107^ expanded this approach using different selenourea ligands, leading to the synthesis of five new gold­(I) complexes of general formula [Au­(IPr)­(selenourea)]­PF_6_ (Scheme 4). In the first report, cyclic selenoureas **24** were employed to prepare the corresponding gold­(I) derivatives **25**.

**Scheme 4:**
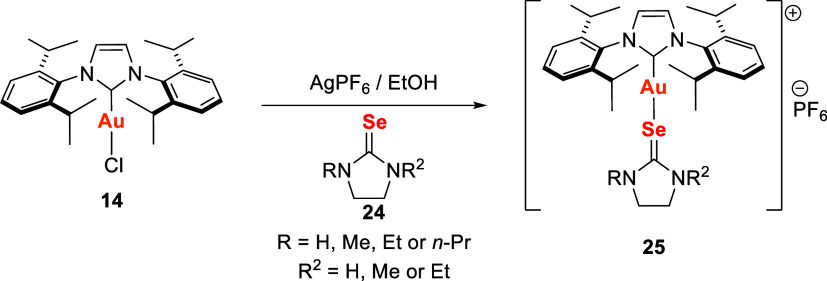
Synthetic Route of Gold­(I) Complexes with NHC Ligands and Different Selenoureas

*In vitro* cytotoxicity was assessed against three human cancer cell lines: A549 (lung carcinoma), HCT15 (colon cancer), and MCF7 (breast cancer). The IC_50_ values indicated that the gold­(I) complexes exhibited cytotoxic activity comparable to cisplatin and superior to their precursor [Au­(IPr)­Cl] (**14**). Notably, complex 25 (R = R_2_ = H) showed the highest potency against HCT15 cells, with an IC_50_ of 33 ± 1 μM.^106^

Subsequent studies expanded the series of selenoureas to compounds **26a–26e** (Figure 7). Docking analyses revealed that complex **26a** had the strongest binding affinity (−38.68 kcal·mol^–1^) for thioredoxin reductase (TrxR), involving interactions with the selenocysteine-containing active site as well as van der Waals, π-cation, and alkyl contacts. Complementary electrochemical studies demonstrated pronounced interactions between the gold complexes, tryptophan, and lysozyme, evidenced by decreased oxidation peak currents and positive shifts in peak potentials. These findings provide mechanistic insights into both the cytotoxicity and target engagement of these complexes.^107^

**Figure 7:**
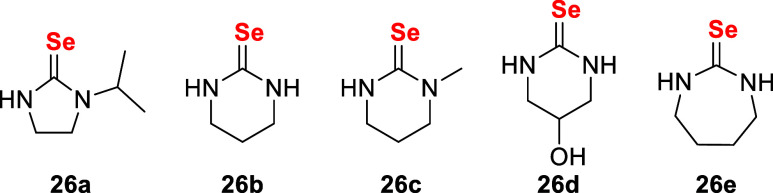
Structures of the selenourea ligands **26a**–**26e**.

Molter et al.^108^ conducted a comparative study between antitumor gold­(I) complexes bearing thio- and selenoureato ligand (Scheme 5). The selenoureato ligand **17** was synthesized by reacting selenourea **19** with AuCl­(PTA) (PTA = 1,3,5-triaza-7-phosphaadamantane) in the presence of sodium methoxide in methanol, yielding a neutral gold­(I) PTA complex with the deprotonated selenoureato ligand. The selenourea precursor **19** was prepared from 4-nitrobenzoyl chloride **18** and KSeCN in a PEG-400/dichloromethane mixture, followed by the addition of diethylamine.

**Scheme 5:**
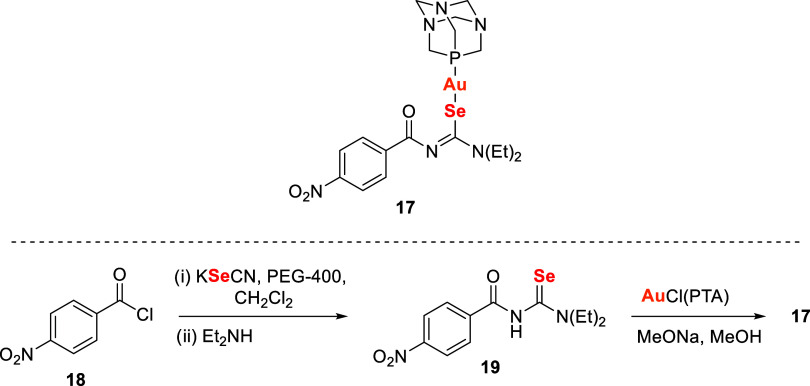
Gold Complexes with Selenoureate Ligand **17**

The *in vitro* activity of these complexes was evaluated in mammary carcinoma (MDA-MB231, MCF-7), ovarian carcinoma (A-2780), acute lymphocytic leukemia (HL-60), and chronic myeloid leukemia (K-562) cell lines. The gold­(I)-selenoureato complex **17** exhibited antiproliferative effects at 9 μM, whereas the corresponding sulfur analogue was active at 18 μM. In all cases, selenium derivatives showed superior activity compared to their sulfur counterparts. Testing in cisplatin-resistant A-2780 cells further highlighted the potential of these complexes to overcome chemotherapy resistance. Mechanistic studies indicated that their effects involve inhibition of proliferation and metabolic activity, alongside induction of apoptosis and oxidative stress through ROS generation.

Beyond anticancer activity, gold complexes have demonstrated antimicrobial properties. Auranofin, for example, shows strong activity against Gram-positive bacteria but limited efficacy against Gram-negative strains.^5,109^ To address this, Chen et al.^110^ synthesized a series of gold­(I) complexes with seleno-substituted N-heterocyclic carbene (Se-NHC) ligands (Scheme 6), in combination with triethylphosphine or triphenylphosphine.

**Scheme 6:**
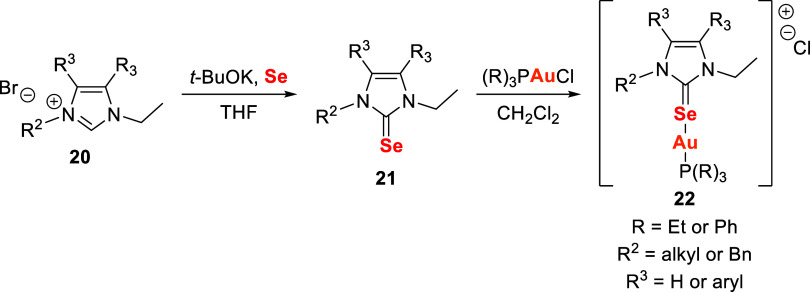
Synthetic Route for the Preparation of Gold Complexes with Seleno-Substituted NHC Ligands **22**

The selenourea intermediates **21** were obtained from the corresponding imidazolium salts **20** by treatment with potassium *t*-butoxide in THF and elemental selenium. Subsequent reaction of **21** with chloro­(triethylphosphine) or chloro­(triphenylphosphine) gold­(I) in dichloromethane afforded the final gold complexes **22**.

These complexes were tested for antibacterial activity against multidrug-resistant (MDR) bacteria, including Acinetobacter baumannii (CRAB), *P. aeruginosa* (CRPA), *K. pneumoniae* (CRKP), and *E. coli* (CREco), all resistant to carbapenems, as well as *S. aureus* with reduced vancomycin susceptibility (VISA). Among the synthesized compounds, **23a** and **23b** (Figure 8) displayed strong activity against MDR strains (MIC = 10–20 μM) and VISA (MIC = 0.2 μM), outperforming auranofin (MIC = 20–40 μM for MDR and 0.2 μM for VISA).^97^ The inactivity of the free ligands confirmed that the antibacterial effect is mediated by the gold center.

**Figure 8:**
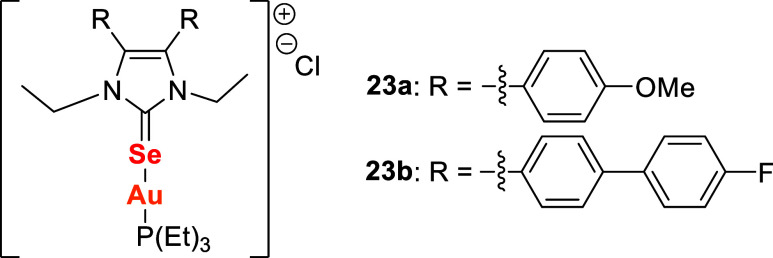
Chemical structures of **23a** and **23b**.

The antibacterial activity of these complexes likely arises from inhibition of the TrxR enzyme, a mechanism also observed for bioactive compounds such as auranofin, ebselen, shikonin, and allicin, which are effective against Gram-positive bacteria.^111^ However, most Gram-negative bacteria possess an additional glutathione (GSH)-based system, contributing to auranofin resistance by destabilizing the complex.

Accounting for this, Chen et al. evaluated the inhibitory effects of auranofin **5** and complexes **23a** and **23b** on CRAB TrxR. Auranofin inhibited the enzyme in a dose-dependent manner (IC_50_ = 6.31 μM), while **23a** and **23b** showed stronger inhibition (IC_50_ = 1.58 μM and 1.20 μM, respectively).^97,110^ The antibacterial activity of these gold­(I) complexes involves irreversible TrxR inhibition via interaction with its redox-active motif, coupled with cellular DNA degradation. By impairing the enzyme’s ability to neutralize reactive oxygen species (ROS), treatment with **23a** and **23b** induced oxidative stress and subsequent metabolic dysfunction.

### 2.3 Gold­(I) with Modified Selenolate

Ott, Menia, and collaborators^112^ reported the development of air-stable homo- and heteroleptic gold­(I) complexes containing the zwitterionic cobaltoceniumselenolate ligand (Scheme 7). The precursor was obtained by treating iodocobaltocenium salt **27** with sodium selenide, prepared in situ from sodium and selenium. Reaction of **28** with 1 equiv of chloro­[(triphenylphosphine)]­gold­(I) yielded complex **29**, while treatment with 0.5 equiv of (Ph)­3PAuCl produced complex **30**.

**Scheme 7:**
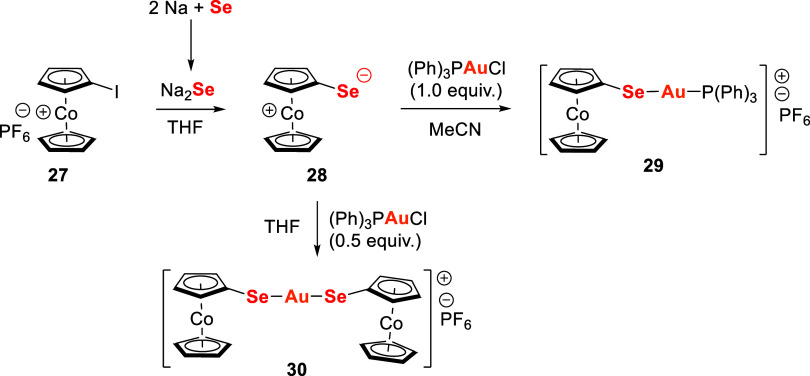
Gold­(I) Complexes with Cobaltoceniumselenolate Ligands **29** and **30**

Compounds **29** and **30** were evaluated for their cytotoxic effects against lung carcinoma (A549), colon adenocarcinoma (HT-29), and breast carcinoma (MDA-MB-231) cell lines. Both complexes demonstrated higher activity than cobaltoceniumselenolate alone, with IC_50_ values ranging from 3.5 to 12.3 μM, confirming that the introduction of the Au­(I) center significantly enhanced their cytotoxic potential.

### 2.4 Gold­(III) and Seleno-Porphyrin

Gold, selenium, and porphyrin are widely used components in anticancer drug design. Yang and collaborators^113^ combined these elements in a Se-modified porphyrin Au­(III) complex, [AuTPP-Se]Cl **34**, as a potential anticancer agent. Synthesis involved the amide bond formation between monomethoxycarbonyl-substituted tetraphenylporphyrin **31** and l-selenomethionine methyl ester **32**, followed by the introduction of Au­(III) through reaction of intermediate **33** with KAuCl_4_·2H_2_O, sodium acetate in acetic acid, and chloroform (Scheme 8).

**Scheme 8:**
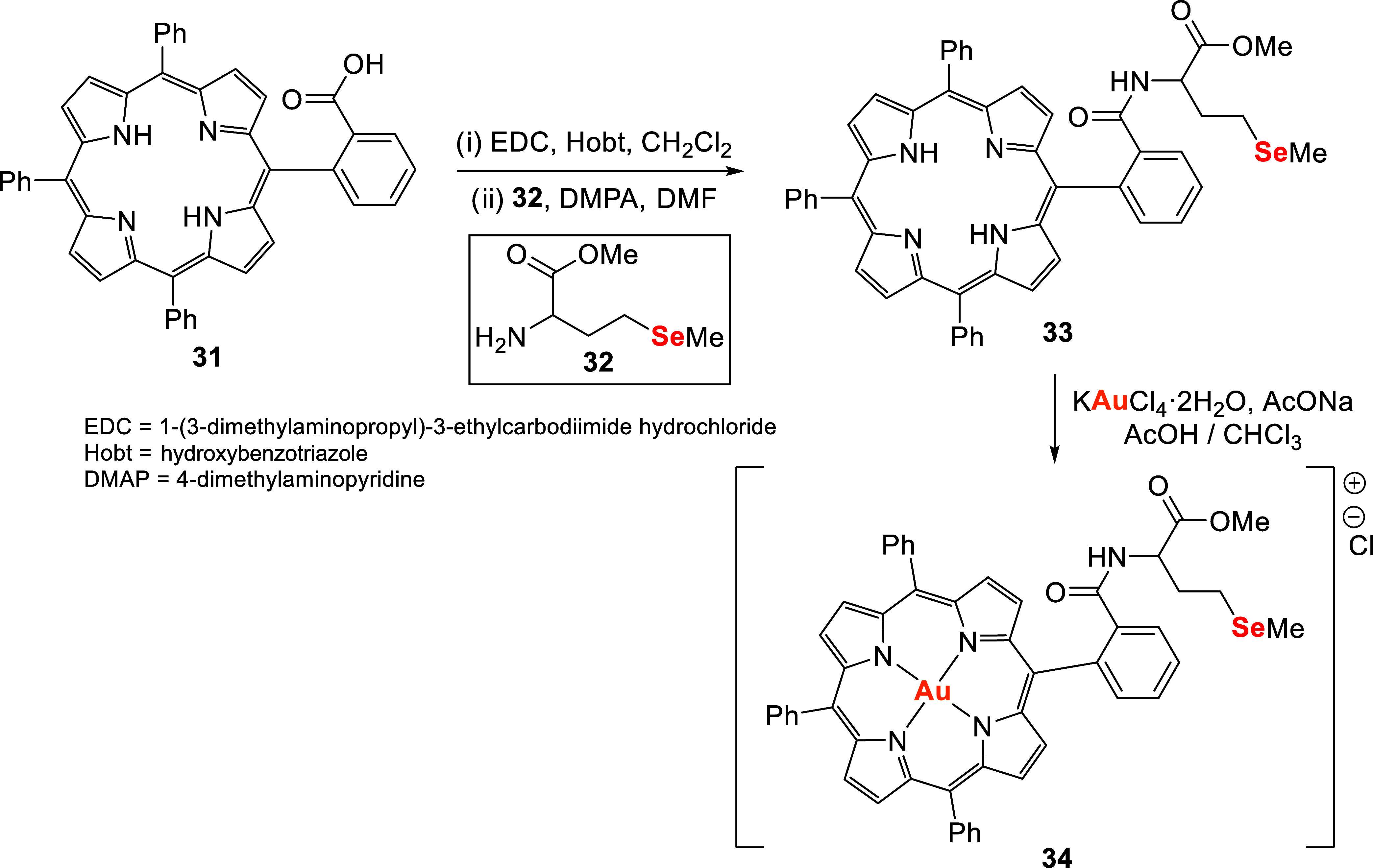
Synthetic Route of [AuTPP-Se]Cl **34**

Compound **34** exhibited remarkable antiproliferative activity across six human cancer cell lines–pulmonary carcinoma (A549), cervical epithelial carcinoma (HeLa), breast carcinoma (MCF-7), erythroleukemia (K562), hepatocellular carcinoma (HepG2), and glioblastoma multiforme (LN229)–with IC_50_ values ranging from 1.42 to 13.65 μM. Notably, it outperformed the selenium-modified porphyrin **33**, the gold–porphyrin complex, and free porphyrin **31**, with IC_50_ values generally lower than those of cis-diaminedichloroplatinum (CDDP). Its potency against HepG2 cells was over ten times higher than CDDP (1.42 μM vs 13.73 μM), highlighting a synergistic effect of Au, Se, and porphyrin.

Mechanistic studies showed that [AuTPP-Se]Cl induced G2 cell cycle arrest, increased intracellular ROS, disrupted mitochondrial function, and modulated Bcl-2 and Bax expression, leading to dose-dependent apoptosis. These findings support its potential as a chemotherapeutic and chemopreventive agent.^113^

### 2.5 Gold­(I) with Selenosemicarbazone

Malaria, caused by *P. falciparum* and transmitted by *Anopheles* mosquitoes, remains one of the deadliest tropical diseases, with over 263 million cases and approximately 597,000 deaths reported in 2023.^114–116^ The emergence of chloroquine resistance emphasizes the urgent need for new chemotherapeutic strategies.

In this context, Molter and collaborators^117^ investigated gold­(I) complexes with thiosemicarbazone and selenosemicarbazone ligands (**36a–36b**) as potential antimalarial agents targeting *P. falciparum*. These complexes were synthesized in a single-step reaction by adding sodium methoxide to a methanol solution of selenosemicarbazone **35a–35b**, followed by the addition of chloro­(triphenylphosphine)­gold­(I) (Scheme 9).

**Scheme 9:**
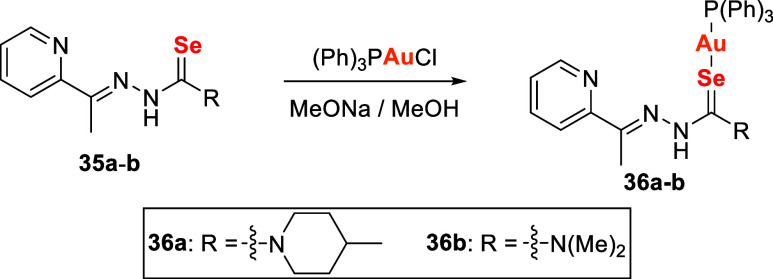
Synthetic Route of Phosphine Gold­(I) Complexes, **36a** and **36b**, Containing Selenosemicarbazones

Both sulfur- and selenium-based ligands enhanced the antimalarial activity of the gold complexes. The sulfur analogue exhibited IC_50_ values comparable to chloroquine (7.06 ± 0.78 nM vs 8.84 ± 2.13 nM), while the selenium derivatives **36a** and **36b** also showed notable activity (IC50 = 60.5 ± 4.45 nM and 135 ± 13.4 nM, respectively). Although less potent than the sulfur analogue, these selenium complexes represent rare examples of Au–Se compounds with antimalarial activity. Their results indicate that selenium incorporation can maintain significant biological efficacy, broadening the therapeutic potential of gold–selenium chemistry in medicinal research.

### 2.6 Gold­(I) with Selenoglucose

The search for new anti-inflammatory agents remains important given the limitations of existing therapies, including reduced efficacy, adverse effects, and poor bioavailability.^118,119^ Gold­(I) complexes have attracted attention in this context due to their distinctive redox-based mechanisms and potential therapeutic applications beyond rheumatoid arthritis.^111^ Within this framework, Hill and collaborators^120^ synthesized a selenium analogue of auranofin (Se-AF) **39** to evaluate the effects of sulfur-to-selenium substitution on the reactivity and pharmacological profile of gold­(I) complexes.

The synthesis involved reaction of acetobromoglucose **37** with selenourea **15** in acetone under reflux, yielding the hydrobromide intermediate **38**. The final gold­(I) complex **39** was obtained in two steps: neutralization of **38** with potassium carbonate in water, followed by addition of (Et)­3PAuCl in a mixture of ethanol and dichloromethane (Scheme 10).

**Scheme 10:**
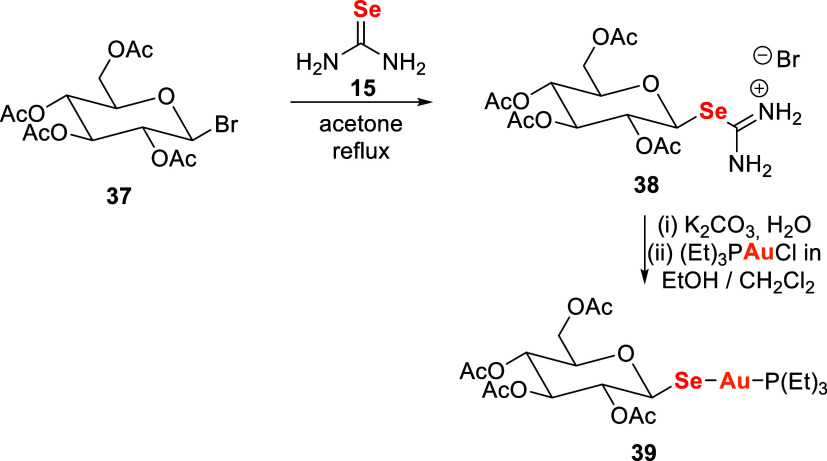
Synthesis of the Selenium Analogue of Auranofin, Se-AF **39**

Complex **39** exhibited increased reactivity in solution, with faster exchange with biomolecules such as human serum albumin (HSA) and more rapid formation of Et3PO compared to auranofin **5** and related gold complexes^121,122^ , reflecting the distinct behavior of the selenolate ligand. In topical inflammation models, **39** showed activities comparable to auranofin, demonstrating that selenium substitution can preserve anti-inflammatory effects under localized conditions. In systemic administration (carrageenan-induced paw edema), however, **39** showed limited efficacy, likely due to rapid metabolism and formation of more polar, less bioavailable metabolites.Overall, this study provided a direct comparison between auranofin and its selenium analogue, offering insights into how sulfur-to-selenium substitution affects stability, metabolism, and pharmacological outcomes in gold­(I) anti-inflammatory agents. The findings highlight Se-AF as a promising tool for further investigation.

## 3 Conclusion and Future Perspectives

This review provides the first focused overview of biologically active selenium–gold complexes, a field that, although still limited in scope, is beginning to reveal distinctive chemical and therapeutic potential. By combining the chemical versatility of gold (e.g., its ability to interact with a variety of enzymes and proteins, enabling distinct mechanisms of action) with the unique redox properties of selenium, these compounds stand out as promising candidates for the development of next-generation metallodrugs. Current studies demonstrate that Au–Se complexes can exert anticancer, antimicrobial, and anti-inflammatory effects. These activities are primarily linked to the inhibition of thiol- and selenol-containing enzymes, disruption of redox homeostasis, and induction of ROS production. The synergistic interplay between the gold and selenium centers emerges as a central element in modulating these biological responses.

Despite these promising findings, the number of structurally and biologically characterized Au–Se complexes remain relatively small when compared to their sulfur analogues. Critical questions regarding stability, selectivity, pharmacokinetics, and long-term safety are still largely unexplored. Moreover, most available studies remain at the in vitro level, underscoring the urgent need for in vivo validation and translational approaches. Addressing these limitations will require (i) broadening the chemical scope of selenium ligands beyond classical selenium ligands, such as selenolates and selenoureas, (ii) systematic structure–activity relationship studies, and (iii) deeper mechanistic investigations of gold–selenium interactions with biomolecular targets such as TrxR, GPx, and mitochondrial proteins.

Future progress in the field depends on further advances in drug delivery and formulation. Strategies to improve solubility, stability under physiological conditions, and targeted delivery are crucial to reduce systemic toxicity while maximizing therapeutic efficacy. In this regard, approaches including prodrug design, as well as nanotechnology and biomolecule conjugation, could represent promising directions. Furthermore, the higher nucleophilicity and lipophilicity conferred by selenium compared to sulfur may provide unique opportunities to exploit hydrophobic enzyme pockets and cellular membranes, which could be leveraged to design compounds with improved selectivity and bioavailability.

Taken together, selenium–gold complexes represent a pioneering and yet underdeveloped class of metallodrugs, with potential to expand the scope of gold-based therapeutics well beyond traditional Au–S systems. By integrating advances in ligand design, drug delivery technologies, and mechanistic understanding, this field has the potential to broaden the scope of gold-based therapeutics, fostering the development of innovative treatments targeting a range of diseases, including cancer, infectious disorders, and inflammatory conditions.

## Abbreviations

A-2780: ovarian carcinoma cell line
A549: pulmonary carcinoma cell line
AF: auranofin
CDDP: cis-diaminedichloroplatinum
CRAB: carbapenem-resistant *Acinetobacter baumannii*
CREco: carbapenem-resistant *Escherichia coli*
CRKP: carbapenem-resistant *Klebsiella pneumoniae*
CRPA: carbapenem-resistant *Pseudomonas aeruginosa*
Et_3_P: triethylphosphine
FDA: Food and Drug Administration
GPx: glutathione peroxidase
GR: glutathione reductase
HeLa: cervical epithelial carcinoma cell line
HepG2: hepatocellular carcinoma cell line
HL-60: acute lymphocytic leukemia cell line
HSA: Human Serum Albumin
IC_50_: half maximal inhibitory concentration
IPr: 1,3-bis­(2,6-diisopropylphenyl)­imidazol-2-ylidene
K-562: chronic myeloid leukemia cell line
LN229: glioblastoma multiforme cell line
MDA-MB231: triple-negative breast carcinoma cell line
MCF-7: estrogen receptor-positive breast carcinoma cell line
MDR: multidrug resistant
NHC: *N*-heterocyclic carbene
NCDs: noncommunicable diseases
NRF2: Nuclear Factor Erythroid 2-related Factor 2
RA: rheumatoid arthritis
ROS: reactive oxygen species
SeC: selenocysteine
SeMet: selenomethionine
Trx: thioredoxin
TrxR: thioredoxin reductase
VISA: vancomycin-intermediate *Staphylococcus aureus*
